# Desiccation Tolerance in the Tardigrade *Richtersius coronifer* Relies on Muscle Mediated Structural Reorganization

**DOI:** 10.1371/journal.pone.0085091

**Published:** 2013-12-31

**Authors:** Kenneth Agerlin Halberg, Aslak Jørgensen, Nadja Møbjerg

**Affiliations:** Department of Biology, August Krogh Centre, University of Copenhagen, Copenhagen, Denmark; University of Colorado, Boulder, United States of America

## Abstract

Life unfolds within a framework of constraining abiotic factors, yet some organisms are adapted to handle large fluctuations in physical and chemical parameters. Tardigrades are microscopic ecdysozoans well known for their ability to endure hostile conditions, such as complete desiccation – a phenomenon called anhydrobiosis. During dehydration, anhydrobiotic animals undergo a series of anatomical changes. Whether this reorganization is an essential regulated event mediated by active controlled processes, or merely a passive result of the dehydration process, has not been clearly determined. Here, we investigate parameters pivotal to the formation of the so-called "tun", a state that in tardigrades and rotifers marks the entrance into anhydrobiosis. Estimation of body volume in the eutardigrade *Richtersius coronifer* reveals an 87 % reduction in volume from the hydrated active state to the dehydrated tun state, underlining the structural stress associated with entering anhydrobiosis. Survival experiments with pharmacological inhibitors of mitochondrial energy production and muscle contractions show that i) mitochondrial energy production is a prerequisite for surviving desiccation, ii) uncoupling the mitochondria abolishes tun formation, and iii) inhibiting the musculature impairs the ability to form viable tuns. We moreover provide a comparative analysis of the structural changes involved in tun formation, using a combination of cytochemistry, confocal laser scanning microscopy and 3D reconstructions as well as scanning electron microscopy. Our data reveal that the musculature mediates a structural reorganization vital for anhydrobiotic survival, and furthermore that maintaining structural integrity is essential for resumption of life following rehydration.

## Introduction

Anhydrobiosis is defined as a reversible entry into a latent state of life in response to desiccation [1]. This phenomenon is widespread across life kingdoms; among animals it is known from rotifers, nematodes and tardigrades as well as certain species of arthropods [2]. In the anhydrobiotic state, metabolic activities come to a reversible standstill, and the organism displays an increased resistance to physiochemical extremes [3]. Tardigrades are microscopic ecdysozoans [4-7] that can remain in this dehydrated state for up to 20 years [8], yet once external conditions again become favorable they resume life unaffected [9-11]. Many anhydrobiotic organisms are known to rely on specific bioprotectants, such as certain saccharides and proteins as well as antioxidant enzymes, in order to offset the damages associated with complete desiccation, e.g. [12-19]; however, a unifying theory on how “life without water” is biologically feasible can still not be claimed. 

 Upon sensing an as yet unidentified cue associated with a decrease in external water potential, anhydrobiotic animals undergo a series of anatomical changes. Rotifers and tardigrades contract in the anterior-posterior direction, and their extremities invaginate, resulting in a compact body shape called a “tun” [20,21]. Nematodes, incapable of a corresponding longitudinal contraction, coil into a tight spiral [20]. The functional significance of these changes has been suggested to be a reduced rate of evaporative water-loss, as well as a controlled packaging of organs, cells and organelles during the desiccation process [21-24]. Studies on anhydrobiotic rotifers [21,25] and nematodes [26] suggest that this reorganization of internal anatomy is coordinated and necessary for maintaining structural integrity and for anhydrobiotic survival. However, experiments on the desiccation tolerant larvae of the sleeping chironomid, *Polypedilum vanderplanki*, indicate that anhydrobiosis may proceed without coordination from the central nervous system [27]. As such, the question of whether this reorganization is a vital regulated event, mediated by active controlled processes, or merely a passive result of the dehydration process, remains unclear.

 Here, we investigate the anatomical changes that occur during anhydrobiosis in the tardigrade *Richtersius coronifer* (Richters, 1903), a species well known for its ability to enter anhydrobiosis [10,28,29]. We show that mitochondrial energy production and a functional musculature are prerequisites for the formation of the tun state. We furthermore present a detailed analysis of the musculature involved in tun formation. 

## Materials and Methods

### Ethics Statement

**Figure 1:**
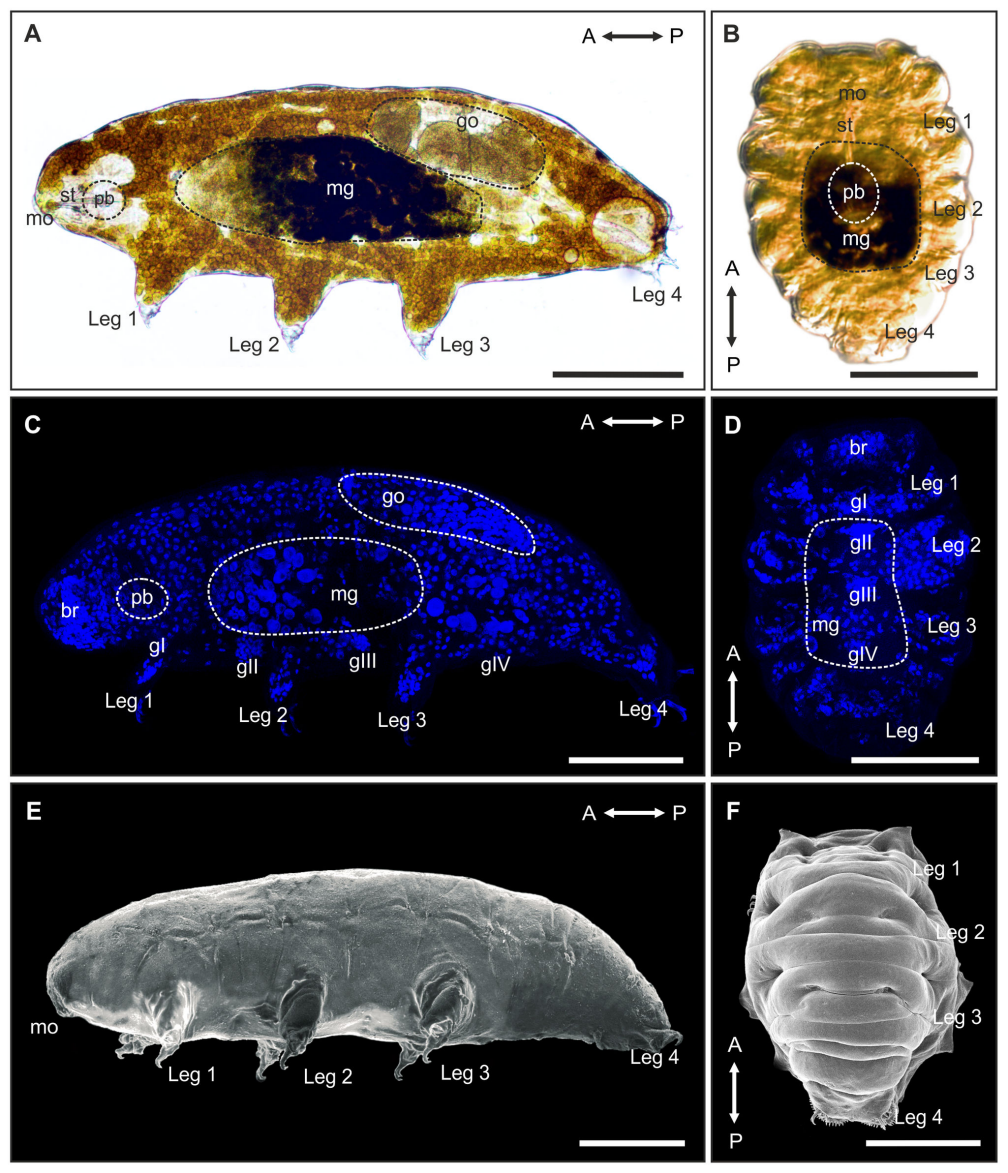
Rearrangement of organs and cells during anhydrobiotic tun formation in *Richtersius coronifer*. Light microscopy of **A**. active, hydrated animal (lateral view) and **B**. a tun (ventral view) showing the rearrangement of major anatomical structures during tun formation. Note the compact body shape of the tun. Dashed circles indicate areas of the midgut (mg), gonads (go) and pharyngeal bulb (pb), respectively. The degree of longitudinal contraction is ultimately limited by the length of the rigid stylets (st). The pharyngeal bulb is for the most part repositioned in the dorsomedian plane. Maximum projection image of a confocal z-series of **C**. hydrated DAPI stained specimen (lateral view), and D. DAPI stained tun (ventral view) demonstrating the reposition of cell nuclei during tun formation. Scanning electron micrograph of **E**. a hydrated specimen (lateral view) and **F**. a tun (dorsal view) revealing the extensive changes in external morphology associated with formation of the tun. A↔P, anterior-posterior axis; br, brain; gI-gIV, ventral ganglia; mo, mouth. Scale bars = 100 μm.

Specimens of the tardigrade *Richtersius coronifer* (Figure 1) were collected from mosses on Öland, Sweden. Collection of specimens was approved by Station Linnè (Porten till Alvaret).

### Storing of tardigrades

Active animals were sorted from water soaked moss using a dissection microscope, and kept in ddH_2_O at 4 °C for two to three days to ensure that they remained active. Groups of 20-25 tardigrades, cleaned of debris, were transferred to, and dehydrated on small pieces of Whatman 3 filters (diameter app. 5 mm; see 28). Filters with dehydrated *Richtersius coronifer* tuns were mounted on microscope glass slides and stored at 4 °C, for a maximum of 2 weeks, until experimentation. 

### Measurements of body volume

The volume of hydrated animals (Figure 1A, 1C, 1E) was estimated as a cylinder (*V*=*πr* ^2^ *h*), while the volume of dehydrated animals (Figure 1B, 1D, 1F) was estimated as a hemicylinder ($V=\frac{\pi r^{2}h}{2}$), in which *V* is the volume of the animal, *r* is half the measured width and *h* is the measured length. The length (μm) of both hydrated and dehydrated animals was defined as the length from the anterior tip to the junction between the fourth leg pair, whereas width (μm) was measured between leg two and three. Measurements were performed using the image software DP-soft^TM^ (Olympus, Germany).

### Exposure to toxins

In order to test if tun formation is an active process, or alternatively a passive, secondary effect coupled to loss of body water, we investigated how pre-incubation in the mitochondrial uncoupler 2, 4-dinitrophenol (DNP; see e.g. 30) affects anhydrobiotic survival in *Richtersius coronifer*. DNP concentrations were used in a range known to work on tardigrade epithelia [31]. Filters containing groups of approximately 20 specimens of dehydrated *R. coronifer* were rehydrated in ddH_2_O water approximately 24 h prior to experimentation, and only animals that resumed activity were used for further experimentation. Experiments ran for a total of 5 days, each ending with a period of 72 or 96 h (depending on experimental procedure), in which animal survival was assessed. For each DNP concentration tested, three groups were prepared in parallel, in which two groups were incubated in DNP and one group was incubated in ddH_2_O for 24 h at 4 °C. Following the incubation period, one of the groups incubated in DNP, and the one kept in ddH_2_O were dehydrated on Whatman filters at 22 °C at ambient relative humidity, and then stored at 4 °C. The dehydration process, from fully hydrated to completely desiccated (see Movie S1), was completed within 30 minutes. The last group incubated in DNP was rinsed several times in ddH_2_O, and subsequently stored at 4 °C. After 24 h, both dehydrated groups were rehydrated, and allowed to revive over a further 72 h. One additional group kept in ddH_2_O during the five-day experimental period provided an estimate of baseline mortality. Survival was subsequently assessed for all groups with animals responsive to tactile stimuli being considered alive. Concentrations of 0.1 mM and 1.0 mM DNP were tested, with four to five experimental repeats conducted for each concentration. 

We further investigated how pre-incubation in unlabeled phalloidin affected anhydrobiotic survival in *Richtersius coronifer*. Phalloidin is a bicyclic heptapeptide that selectively binds and stabilizes actin filaments (F-actin), which blocks nucleotide exchange [32] and consequently inhibits cross-bridge cycling and muscle contractions. Preliminary phalloidin incubation experiments, using fluorescent phalloidin, revealed that the primary entry site of the toxin was through the mouth and cloaca of the tardigrade. In these experiments, phalloidin would similarly be observed staining the muscles, as visualized by the fluorescent signal (data not shown), thus confirming that the toxin had access to the muscles during incubation experiments. The experimental procedure for pre-incubating animals in unlabeled phalloidin was as described above for the DNP-experiments, but with concentrations of 0.01, 0.1, 0.5 and 1.0 mg/ml phalloidin tested instead of DNP. Five experimental repeats were conducted at each concentration.

**Figure 2:**
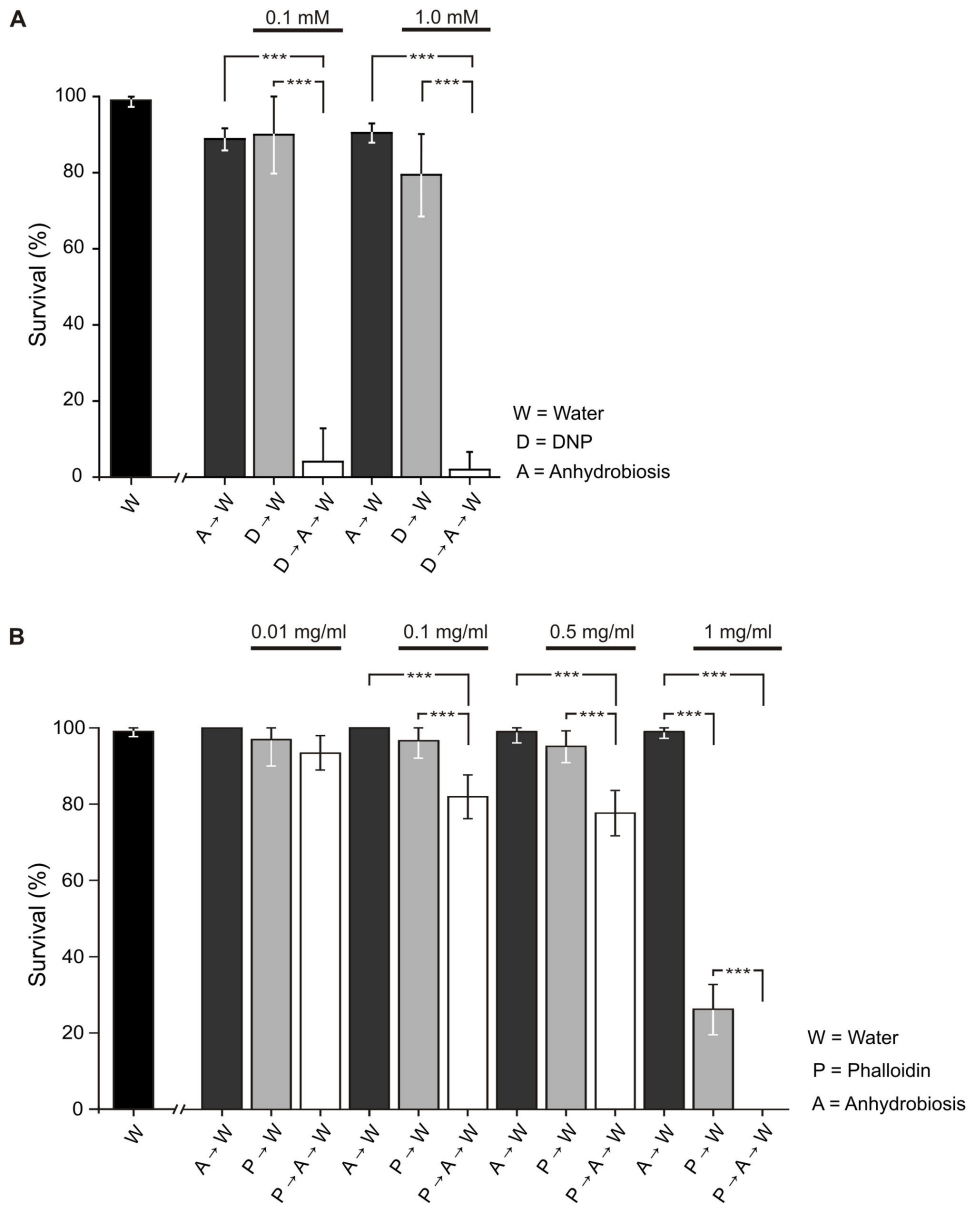
Effect of DNP and phalloidin on anhydrobiotic survival. **A**. Pre-incubation in DNP prior to dehydration, and attempting to induce anhydrobiosis (D→A→W), significantly reduces survival to 4 ± 9 % (0.1 mM) and 2 ± 4 % (1.0 mM). **B**. Incubating tardigrades in phalloidin (P→W) did not decrease survival at 0.01 mg/ml (97 ± 7 %), 0.1 mg/ml (96 ± 4 %) and 0.5 (95 ± 5 %) mg/ml. At 1 mg/ml, survival was significantly reduced to 26 ± 7 % (*P*<0.001; Table S2). Pre-incubation in phalloidin (P→A→W) reduced post-anhydrobiotic survival at concentrations of 0.1 mg/ml (82 ± 6 %), 0.5 mg/ml (77 ± 6 %) and 1.0 mg/ml (0 ± 0%) (*P*<0.001; Table S2). Significant differences between treatments are indicated by asterisks, with the significance levels *P*>0.05 (not significant) and *P*≤0.001 (significant, ***).

Following the dehydration protocol described above, and excluding animals damaged during placement on Whatman filters, an average of 97 ± 5 % animals survived induction of anhydrobiosis based on all the control experiments (A→W, Figure 2A-B). This notable survival rate, which is comparable to that reported previously [28], is not significantly different from the baseline survival, i.e. animals kept in ddH_2_O (W, Figure 2A-B; Tables S1, S2), demonstrating that anhydrobiosis is not associated with increased mortality in *Richtersius coronifer* using this protocol. 

### Microscopy

**Figure 3:**
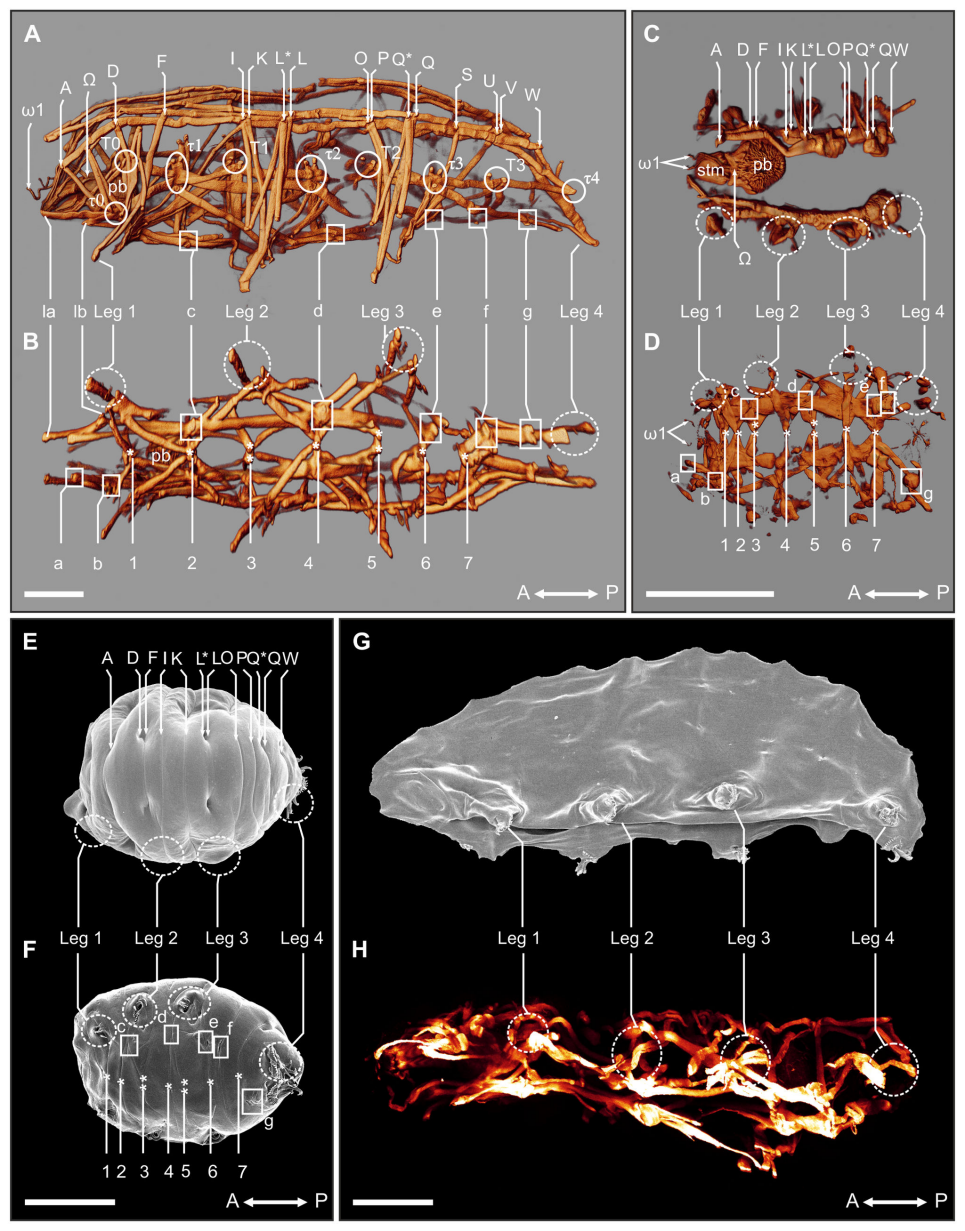
Myoanatomical changes in *Richtersius coronifer* during tun formation. **A**-**D**. 3D reconstructions of the tardigrade musculature as visualized by fluorescent phalloidin. **A**. Lateral view of active (hydrated) state showing details of the dorsal, lateral and leg musculature. **B**. Ventral view showing details of the ventral longitudinal musculature in the active state. **C**. Dorsal view of the myoanatomy of the tun (dehydrated) state D. Ventral view of the myoanatomy of the tun. **E**-**F**. Scanning electron microscopy (SEM) of animals in the tun state showing the corresponding external morphology of the myoanatomy presented in C and D. **E**. Dorsal view of the tun. **F**. Ventral view of the tun. **G**. SEM of an animal incubated in 1.0 mg/ml phalloidin for 24 h and subsequently dehydrated. The animal failed to form a tun upon dehydration, and collapsed into a flattened shape. A similar collapse was seen in DNP exposed animals upon dehydration. **H**. Corresponding maximum projection image of a confocal z-series of the musculature of a specimen incubated in 1.0 mg/ml phalloidin for 24 h before dehydration. A↔P, anterior-posterior axis; A-W, dorsal attachment sites; τ_0_-τ_4_, lateral attachment sites; la-g, ventral intermediate attachment sites; 1-7, ventromedian attachment sites; pb, pharyngeal bulb; ω1-Ω, attachment sites of muscles associated with the pharyngeal bulb; stm, stylet muscles. Solid circles indicate lateral attachment sites, solid squares show ventral intermediate attachment sites, while dashed circles indicate areas of the legs. Scale bars = 100 μm.

Fluorescent labeling of muscles and cell nuclei were performed in order to investigate morphological changes (e.g. rearrangement of organs and cells) occurring during anhydrobiosis in *Richtersius coronifer*. Active tardigrades were relaxed using CO_2_-enriched water, whereas dehydrated specimens placed on Whatman filters were “dry fixed” (i.e. placed over the fumes of a 3 % paraformaldehyde fixative) for 30 min, prior to fixation. Both hydrated and dehydrated animals were subsequently fixed for 20 min at RT in 3 % paraformaldehyde in 0.1 mol/l sodium cacodylate buffer (pH 7.4). For F-actin staining, animals were exposed to 90 seconds of ultrasonication (Branson 2210, Branson Ultrasonics, Netherlands) and incubated in PBS with 1 % Triton X-100, 0.1 % NaN_3_ and a 1:20 dilution of Alexa Fluor 488 conjugated phalloidin (Invitrogen, CA, USA) for up to 48 h. Afterwards, the specimens were rinsed three times in PBS. Additional preparations were stained with DAPI (20 μg/ml) in order to visualize nuclei. Specimens were mounted on microscope slides in Vectashield (Vector Laboratories Inc., CA, USA). Image acquisition was performed on a Leica DM RXE 6 TL inverted microscope equipped with a Leica TCS SP2 AOBS confocal laser scanning unit, using the 488 nm argon/crypton laser or the 405 nm UV-laser. A maximum projection or normal shading of the z-series image was processed and edited in the 3D reconstruction software IMARIS (Bitplane AG, Zürich, Switzerland). All confocal images are based on 140-160 optical sections of a z-series performed at intervals of 0.5 µm. Three active, four anhydrobiotic and two ‘collapsed’ specimens, which had failed to form proper tuns, formed the basis for the descriptions (see Figure 3). 

In order to visualize the external morphology of *Richtersius coronifer*, we used scanning electron microscopy. Active specimens were relaxed in CO_2_-enriched water and subsequently fixed in 2.5% glutaraldehyde in 0.1 M sodium cacodylate buffer (pH 7.4). Following fixation, the specimens were dehydrated through a graded series of ethanol and acetone. The specimens were then critical point dried (Bal-Tec CPD 030 critical point dryer), mounted on aluminum stubs, sputter-coated with palladium (65-70 s corresponding to a thickness of app. 12 nm; JEOL JFC-2300 HR sputtercoater) and examined in a JEOL JSM-6335F Field Emission scanning electron microscope. Both anhydrobiotic tuns and dehydrated collapsed specimens were mounted directly on aluminum stubs, sputter-coated with palladium and examined.

### Statistics

Data are expressed as means ± s.d. The statistical significance of differences between the various exposures was tested using one-way ANOVA followed by a Tukey’s multiple comparisons of means (Table S1 and S2). The statistical tests were performed using the data analysis program OriginPro 7.5 (OriginLab, Northampton, MA, USA). Significant difference between treatments is indicated by asterisks, with significance levels being *P*>0.05 (not significant), *P*≤0.05 (significant, *), *P*≤0.01 (significant, **) and *P*≤0.001 (significant, ***).

## Results and Discussion

### Gross morphology

*Richtersius coronifer* is a large tardigrade species measuring up to more than 1000 µm [33]. It has an elongated body outline typical of eutardigrades with few visible sensory appendages, and four pairs of legs each equipped with two double claws (Figure 1A, 1C, 1E). As in other tardigrades, the complex internal organ systems include a large brain and well developed nervous and muscular systems, a complex feeding apparatus with a muscular pharynx (the pharyngeal bulb) and associated stylets for puncturing food particles, a subdivided alimentary tract, as well as reproductive and osmoregulatory organs. The specimens of *R. coronifer* from Öland were yellow and laid round eggs ornamented with heavy spines. 

### Anatomical changes during anhydrobiosis

Anhydrobiotic tardigrades respond to removal of external water by contracting in the anterior-posterior direction, and at the same time withdrawing head and limbs, forming the compact body shape called a tun (Figure 1B, 1D, 1F; Movie S1). According to our observations of *Richtersius coronifer*, this process is initiated when the animal senses a cue associated with change in external water potential. The process can be divided into three separate stages: I) active and hydrated; II) dehydrating and ‘tucking in’; III) anhydrobiotic tun state (see Movie S1). Our estimations of body volume in *R. coronifer* reveal an 87 ± 5 % (n=17) reduction in volume from the hydrated active state to the dehydrated tun state (Table 1). This drastic change in body volume is larger than the 60 % reduction reported from bdelloid rotifers [25] and further underlines the structural stress associated with entering anhydrobiosis. Conversely, hydrated, active specimens of the marine tardigrade *Halobiotus crispae* have been shown to tolerate above 60 % increase in body volume, during exposures to hypotonic solutions, thus emphasizing the amazing ability of tardigrades to withstand physical stress [34,35]. The degree of longitudinal contraction during tun formation in *R. coronifer* varies between individual tardigrades, but is ultimately limited by the length of the rigid stylets (Figure 1A-B). The pharyngeal bulb is for the most part repositioned in the dorsomedian plane of the animal (Figure 1B), its relocation relying on a flexible esophagus. During the longitudinal contraction, and in concert with the evaporative loss of the fluid filled body cavity, organs and cells seem to be tucked in place by undulatory movements of the trunk (stage II – ‘tucking in’, Movie S1; Figure 1C-D). 

**Table 1:** Reduction in volume (%) of *Richtersius coronifer* from active (hydrated) to tun (dehydrated) state.

***N***	**Hydrated**		**Dehydrated**		**Reduction in volume (%)**
	**Length (μm)**	**Width (μm)**	**Length (μm)**	**Width (μm)**	
1	664	223	252	121	94
2	664	220	321	175	85
3	575	205	316	151	85
4	568	183	250	116	91
5	704	203	242	141	92
6	657	203	291	137	90
7	491	170	189	142	87
8	526	162	231	141	83
9	701	213	340	146	89
10	463	185	179	127	91
11	593	211	200	150	91
12	503	175	185	141	88
13	586	204	148	163	86
14	466	161	180	126	88
15	651	128	183	160	78
16	563	212	307	163	84
17	535	210	280	134	89
**Mean ± s.d. reduction in volume (%**)					**87 ± 5**

### Tun formation relies on mitochondrial energy production

Animals exposed to DNP for 24 h became passive and bloated, but regained activity following transfer to double distilled water (D→W, Figure 2A) with only a small decrease in survival, as compared to water controls (W, Figure 2A), at the highest DNP dose (survival decreased to 80 ± 11 %, *P*<0.05; Table S1). However pre-incubating specimens in DNP for 24 h prior to inducing anhydrobiosis almost completely abolished survival (D→A→W, Figure 2A). These DNP exposed animals failed to form a tun and collapsed into an irregular flattened shape upon dehydration (Figure 3G), indicating that successful dehydration and the ability to form a tun is dependent on mitochondrial energy production. This observation is supported by an earlier report stating that eutardigrades, of the species *Paramacrobiotus areolatus*, failed to form tuns under anoxic conditions [22].

### A functional musculature is a prerequisite for anhydrobiotic survival

Incubating animals in respectively 0.01, 0.1 and 0.5 mg/ml phalloidin for 24 h, before transferring them to water (P→W, Figure 2B), did not decrease survival significantly, though survival was reduced at 1 mg/ml (Figure 2B; Table S2). However, pre-incubating animals in 0.1 and 0.5 mg/ml phalloidin, prior to inducing anhydrobiosis, significantly reduced post-anhydrobiotic survival (P→A→W, Figure 2B), indicating that a functional muscle-system is indeed vital for anhydrobiotic survival. No tardigrades survived anhydrobiosis after pre-incubation in 1 mg/ml phalloidin. Notably, animals in which the muscle system was rendered non-functional, collapsed in a manner similarly to the DNP-treated animals upon drying, and did not revive following rehydration. 

We subsequently investigated the myoanatomy of *Richtersius coronifer* in active, tun and dehydrated collapsed states with the aid of fluorophore*-*conjugated phalloidin (Figure 3). In tardigrades, the body musculature is composed of structurally independent muscle fibers that can be assigned to ventral, dorsoventral, dorsal, lateral, and leg musculature [36,37]. The ventral musculature of *R. coronifer* is dominated by seven ventromedian attachment sites (labeled 1-7, Figure 3B, 3D, 3F) from which leg muscles, dorsoventral muscles and lateral muscles originate. In addition, a ventral longitudinal musculature with intermediate attachment sites (labeled c-g, Figure 3A-B, 3F) extends along the anterior-posterior axis. The dorsal longitudinal musculature consists of an outer and an inner muscle strand that both extend the length of the trunk. Both strands are repetitively interrupted by attachment sites (labeled A-W, Figure 3A, 3C, 3E) mainly associated with the legs. Nine lateral sites (labeled t0-t4 and T0-T3, Figure 3A) serve as attachments for the lateral musculature and the dorsoventral muscles. Leg muscles in *R. coronifer* originate from the dorsal, lateral and ventral side of the animal (see 36,37 for further information). The confocal images show that the muscles are contracted in the tun state in comparison to the active, hydrated specimens (Figure 3A-D). Animals that were exposed to 1 mg/ml phalloidin, and subsequently dehydrated, collapsed into a flattened shape and revealed a more disordered muscle organization, in which individual structural elements where difficult to recognize (Figure 3G-H). 

### Analysis of the Musculature Involved in Tun Formation

**Figure 4:**
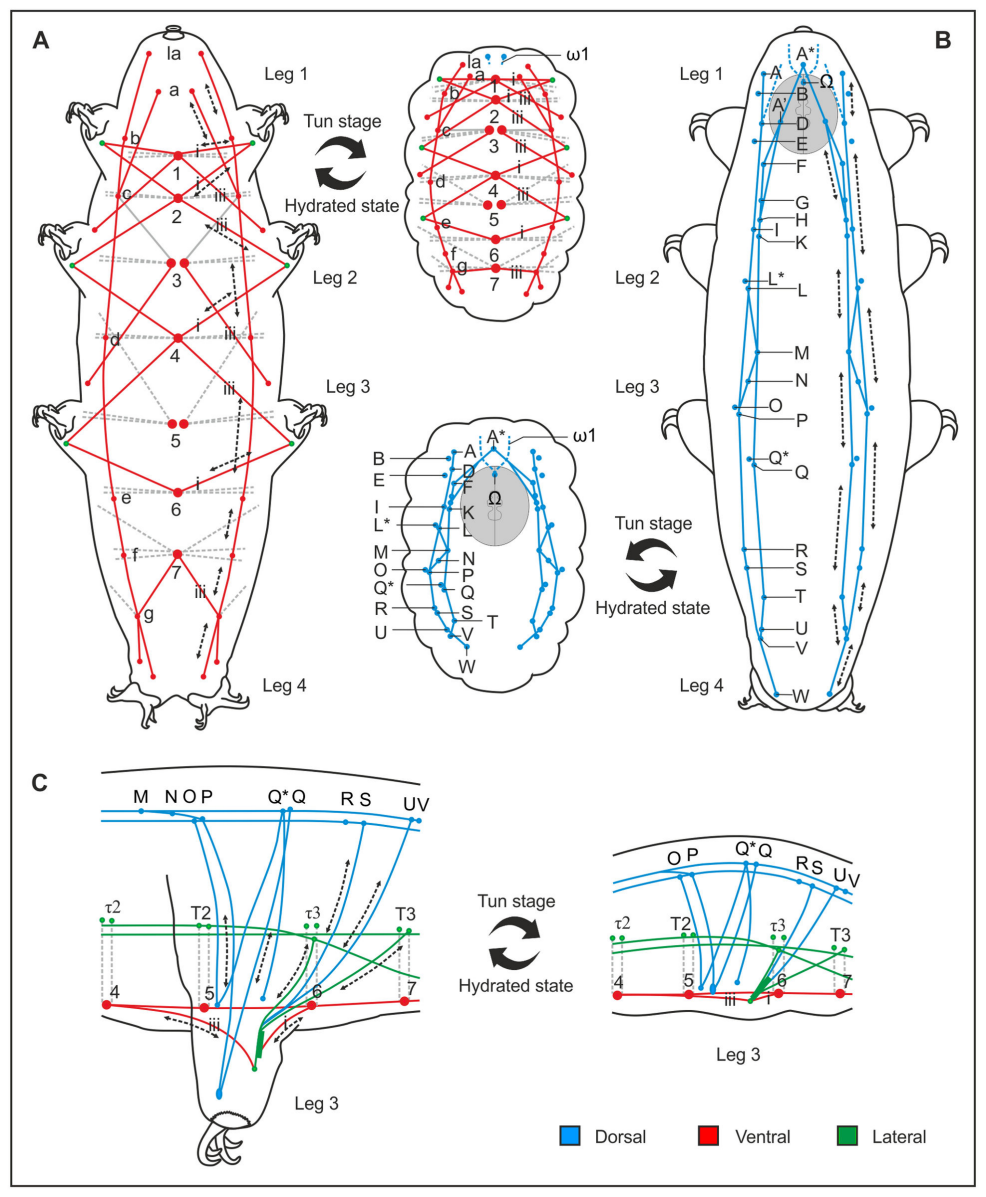
Schematic representation of the muscles involved in tun formation in *Richtersius coronifer*. Schematic representation illustrating contraction of muscles during the transition from the active (hydrated) to the tun (dehydrated) state. **A**. Ventral longitudinal musculature. **B**. Dorsal longitudinal musculature. **C**. Leg musculature. The dorsal longitudinal, ventral longitudinal, as well as lateral musculature are involved in reshaping the whole body during anhydrobiosis, and are consequently responsible for generating the compact body shape of the tun. Tun formation is moreover characterized by the withdrawal of the legs into the body cavity. Letters and numbers indicate specific muscle attachment sites (see Figure 3).

As previously shown in rotifers [21], and also suggested for tardigrades, e.g. [22], our study confirms that proper tun formation is essential for anhydrobiotic survival. Our results show that uncoupling mitochondrial energy production and inhibiting muscle contraction interferes with formation of the anhydrobiotic tun, thereby respectively abolishing and reducing the ability of *Richtersius coronifer* to survive desiccation. We propose that the dorsal and ventral longitudinal muscles are responsible for contraction of the animal during entry into the tun state (Figure 4A-B), while the lateral musculature assists in the longitudinal contraction, and generates undulatory movements of the trunk that facilitate reorganization of internal structures (stage II – ‘tucking in’, Movie S1). Furthermore, the muscles associated with each leg are activated in the withdrawal of the legs during tun formation (Figure 4C). Thus a range of muscles direct – in a predictable and coordinated manner – the structural rearrangements necessary for formation of the tun state.

## Supporting Information

Movie S1 **Anhydrobiotic tun formation in *Richtersius coronifer*.** The most obvious morphological changes associated with tun formation are the anterior-posterior contraction of the trunk and retraction of legs. According to our observations of the behavior of animals during entrance into anhydrobiosis, this process is initiated when the animals sense a decrease in external water potential. Entrance into and exit out of anhydrobiosis can be divided into four separate stages (I, active hydrated; II, dehydrating, ‘tucking in’; III, anhydrobiotic tun state; IV, rehydration) the completion of which is an active process orchestrated by the muscle system. The movie was made using an Infinity X Digital Camera (DeltaPix, Denmark) mounted on a Leica MZ 16 microscope (x80 magnification). High resolution AVI files recorded with the camera software were imported into Windows Movie Maker for the creation of the final video sequence.
(WMV)

Table S1 **Statistical analyses of the DNP data.**
(DOC)

Table S2 **Statistical analyses of the phalloidin data.**
(DOC)
