# Supplementary material for: Desiccation Tolerance in the Tardigrade Richtersius coronifer Relies on Muscle Mediated Structural Reorganization
Source: PLoS One. 2013 Dec 31;8(12):e85091. doi: 10.1371/journal.pone.0085091 (PMC3877342; doi:10.1371/journal.pone.0085091)
Supplement: Table S1 — Statistical analyses of the DNP data. (DOC) [file pone.0085091.s002.doc]

**Table S1. Statistical analyses of the DNP data.**

| **DNP (mM)** | |  | **0.1** | | | **1.0** | | |
| --- | --- | --- | --- | --- | --- | --- | --- | --- |
|  |  | **W** | **AW** | **DW** | **DAW** | **AW** | **DW** | **DAW** |
|  | **W** | - | NS | NS | *** | NS | * | *** |
| **0.1** | **AW** |  | - | NS | *** | NS | NS | *** |
|  | **DW** |  |  | - | *** | NS | NS | *** |
|  | **DAW** |  |  |  | - | *** | *** | NS |
| **1.0** | **AW** |  |  |  |  | - | NS | *** |
|  | **DW** |  |  |  |  |  | - | *** |
|  | **DAW** |  |  |  |  |  |  | - |

*F* = 170.18, *P* < 0.001, df = 23, *N* = 30

Significant differences between the exposures depicted in Figure 2A were tested using one-way analysis of variance (ANOVA) followed by Tukey’s multiple comparisons of means with significance levels of *P*≤0.05 (significant, *), *P*≤0.01 (significant, **) and *P*≤0.001 (significant, ***). *P*>0.05 (not significant, NS). The statistical tests were performed using the data analysis program OriginPro 7.5 (OriginLab, Northampton, MA, USA). DNP was used at concentrations of 0.1 mmol/l and 1 mmol/l. W: H_2_O (5 days); AW: H_2_O (24 h), anhydrobiosis (24 h), H_2_O (72 h); DW: DNP (24 h), H_2_O (96 h); DAW: DNP (24 h), anhydrobiosis (24 h), H_2_O (72 h).
