# Supplementary material for: Desiccation Tolerance in the Tardigrade Richtersius coronifer Relies on Muscle Mediated Structural Reorganization
Source: PLoS One. 2013 Dec 31;8(12):e85091. doi: 10.1371/journal.pone.0085091 (PMC3877342; doi:10.1371/journal.pone.0085091)
Supplement: Table S2 — Statistical analyses of the phalloidin data. (DOC) [file pone.0085091.s003.doc]

Table S2. Statistical analyses of the phalloidin data.

| **Phalloidin (mg/ml)** | |  | **0.01** | | | **0.1** | | | **0.5** | | | **1.0** | | |
| --- | --- | --- | --- | --- | --- | --- | --- | --- | --- | --- | --- | --- | --- | --- |
|  | | **W** | **AW** | **PW** | **PAW** | **AW** | **PW** | **PAW** | **AW** | **PW** | **PAW** | **AW** | **PW** | **PAW** |
|  | **W** | - | NS | NS | NS | NS | NS | *** | NS | NS | *** | NS | *** | *** |
| **0.01** | **AW** |  | - | NS | NS | NS | NS | *** | NS | NS | *** | NS | *** | *** |
|  | **PW** |  |  | - | NS | NS | NS | ** | NS | NS | *** | NS | *** | *** |
|  | **PAW** |  |  |  | - | NS | NS | ** | NS | NS | *** | NS | *** | *** |
| **0.1** | **AW** |  |  |  |  | - | NS | *** | NS | NS | *** | NS | *** | *** |
|  | **PW** |  |  |  |  |  | - | *** | NS | NS | *** | NS | *** | *** |
|  | **PAW** |  |  |  |  |  |  | - | *** | *** | NS | *** | *** | *** |
| **0.5** | **AW** |  |  |  |  |  |  |  | - | NS | *** | NS | *** | *** |
|  | **PW** |  |  |  |  |  |  |  |  | - | *** | NS | *** | *** |
|  | **PAW** |  |  |  |  |  |  |  |  |  | - | *** | *** | *** |
| **1.0** | **AW** |  |  |  |  |  |  |  |  |  |  | - | *** | *** |
|  | **PW** |  |  |  |  |  |  |  |  |  |  |  | - | *** |
|  | **PAW** |  |  |  |  |  |  |  |  |  |  |  |  | - |

*F* = 285.78, *P* < 0.001, df = 52, *N* = 65

Significant differences between the different exposures depicted in Figure 2B were tested using one-way analysis of variance (ANOVA) followed by Tukey’s multiple comparisons of means with significance levels of *P*≤0.05 (significant, *), *P*≤0.01 (significant, **) and *P*≤0.001 (significant, ***). *P*>0.05 (not significant, NS). The statistical tests were performed using the data analysis program OriginPro 7.5 (OriginLab, Northampton, MA, USA). Phalloidin was used at concentrations of 0.01 mg/ml, 0.1 mg/ml, 0.5 mg/ml and 1 mg/ml. W: H_2_O (5 days); AW: H_2_O (24 h), anhydrobiosis (24 h), H_2_O (72 h); PW: phalloidin (24 h), H_2_O (96 h); PAW: phalloidin (24 h), anhydrobiosis (24 h), H_2_O (72 h).
